# Supplementary material for: Determining Antiradical Capacity of Medicinal Plant Extract Individual Constituents Using Post-Column Reaction Method
Source: Int J Mol Sci. 2024 May 17;25(10):5461. doi: 10.3390/ijms25105461 (PMC11122392; doi:10.3390/ijms25105461)
Supplement: Supplementary file 1 [file ijms-25-05461-s001.zip › ijms-2901968-supplementary.pdf]

Determining Antiradical Capacity of Medicinal Plant Extract Individual Constituents Using Post-Column Reaction Method

Jarosław L. Przybył *, Jan Stefaniak, Anna Jaroszewicz, Amanda Gawrońska, Marcin Łapiński, Katarzyna Barbara Bączek and Zenon Węglarz

Department of Vegetable and Medicinal Plants, Institute of Horticultural Sciences, Warsaw University of Life Sciences, Nowoursynowska 159, 02-776 Warszawa, Poland; jstefaniakk@gmail.com (J.S.); anna.m.jaroszewicz@gmail.com (A.J.); jestemamanda@gmail.com (A.G.); marcin.lapinski05@gmail.com (M.Ł.); katarzyna_baczek@sggw.edu.pl (K.B.B.); zenon_weglarz@sggw.edu.pl (Z.W.)

* Correspondence: jaroslaw_przybyl@sggw.edu.pl

Supplementary Materials

Table S1. Validation parameters for the HPLC-DAD method used to separate and determine the biologically active compounds characteristic of *S. divaricata* raw material (n=6).

No.	Compound	Precision intra-day (CV %)	Precision inter-day (CV %)	Calibration equation	R^2 (n=6)	Linear range (µg×mL^{-1})	LOD (µg×L^{-1})	LOQ (µg×L^{-1})	Recovery (%)
1	Chlorogenic acid	1.32	1.63	y = 6517.4 x – 12016.6	0.9997	0.39 – 390.21	4.28	14.29	105.5
2	Prim-O-glucosylcimifugin	1.85	2.51	y = x 1954.89 x – 553.94	0.9999	0.37 – 374.40	2.75	9.16	98.54
3	Rosmarinic acid	1.24	2.12	y = 2017.9 x + 1100.4	0.9999	0.43 – 434.02	3.24	10.81	102.6
4	Cimifugin	0.98	1.85	y = 2905.04 x – 1006.88	0.9998	0.51 – 512.95	5.50	18.32	86.91
5	4'-O-beta-glucosyl-5-O-methylvisamminol	1.26	1.70	y = 1975.98 x – 704.35	0.9999	0.42 – 422.40	3.42	11.23	107.51
6	Imperatorin	1.47	1.98	y = 4029.86 x – 684.02	0.9999	0.43 – 463.15	2.66	8.56	103.47

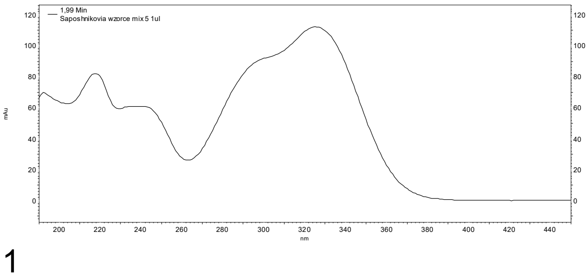

1

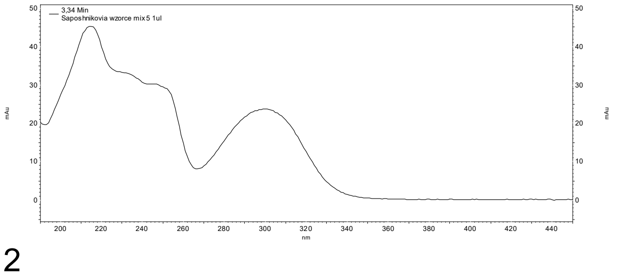

2

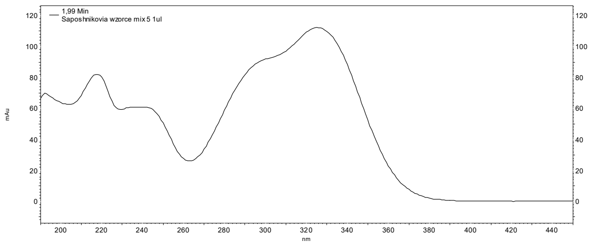

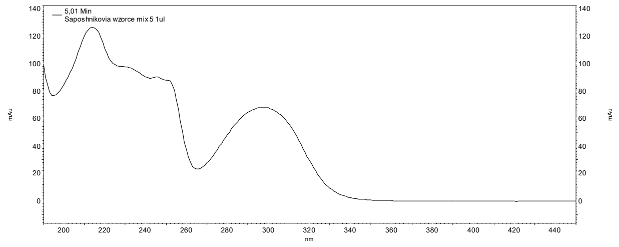

3

4

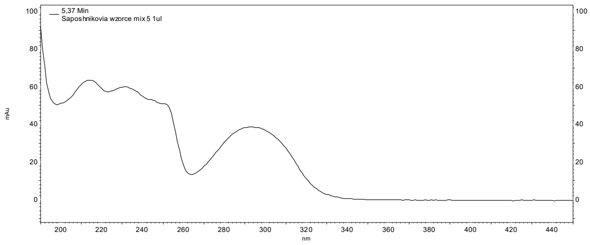

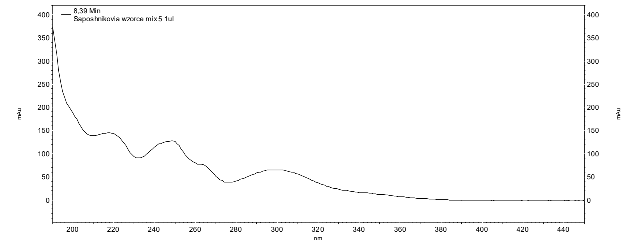

5

6

Figure S1. UV-Vis spectra of 1. Chlorogenic acid (3-O-Caffeoylquinic acid), 2. Prim-O-glucosylcimifugin (Cimifugin β-D-glucopyranoside), 3. Rosmarinic acid, 4. Cimifugin, 5. 4'-O-β-D-glucosyl-5-O-methylvisamminol, 6. Imperatorin (Pentosalen) obtained during this study. Please note that the interpretation of the spectrum in the 190-230 nm range should be approached with caution or abandoned due to the highly probable influence of additional factors.

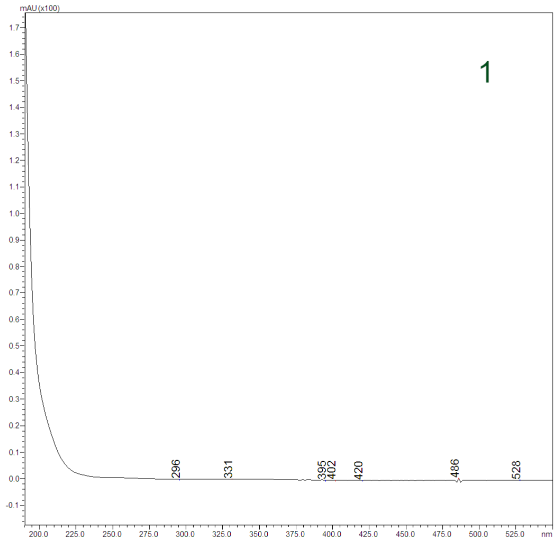

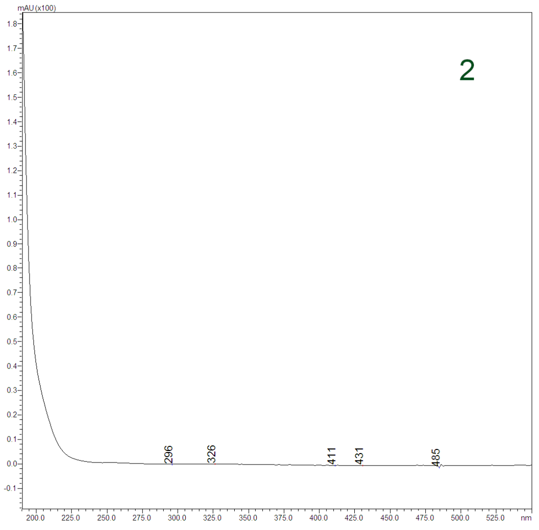

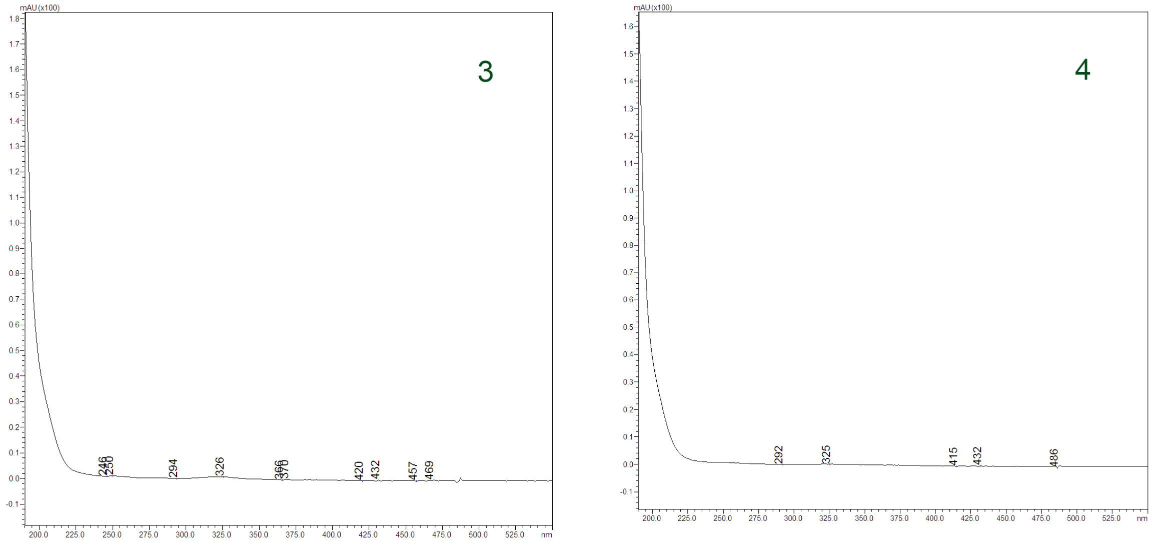

Figure S2. UV-Vis spectra of 1. Astragaloside I, 2. Astragaloside II, 3. Astragaloside III and 4. Astragaloside IV obtained during this study. Please note that the interpretation of the spectrum in the 190-230 nm range should be approached with caution or abandoned due to the highly probable influence of additional factors.

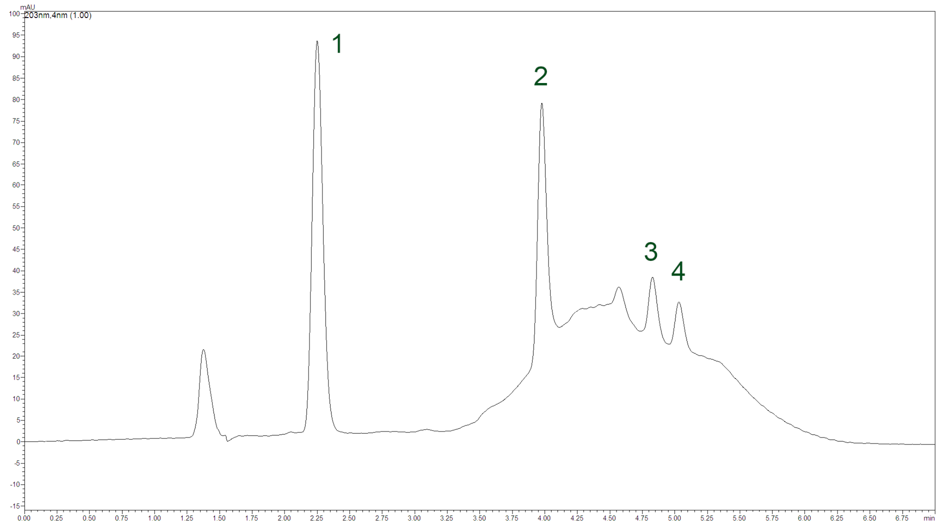

Figure S3. The chromatogram of a mixture of 1. Caffeic acid 2, Rosmarinic acid, 3. Astragaloside I, and 4. Astragaloside III.

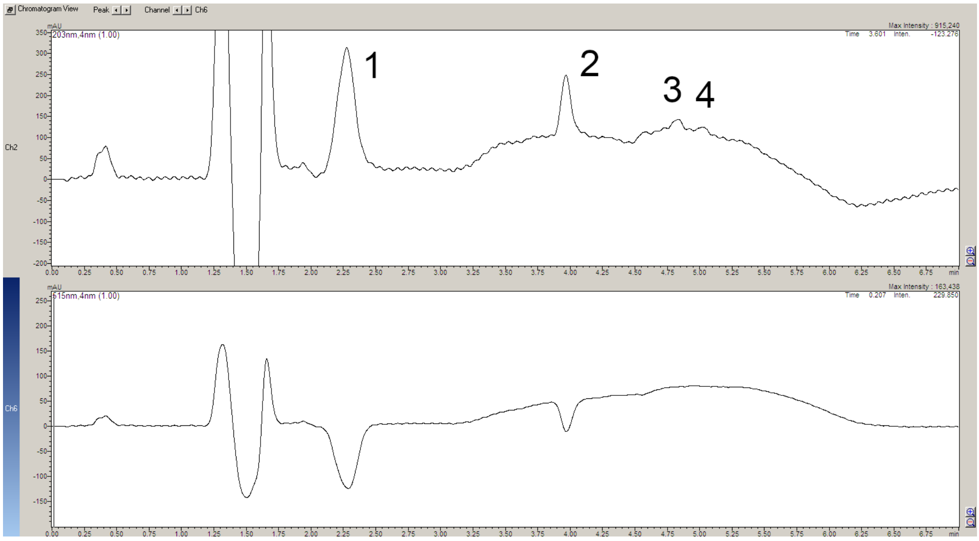

Figure S4. The chromatogram at 203 mn shows the separated compounds present in the methanolic extract of *A. mongholicus* roots along with additionally added caffeic acid and rosmarinic acid (top). The 515 nm chromatogram demonstrates the scavenging of the DPPH● radical by the separated compounds (bottom). Both chromatograms were saved in the same file. 1. Caffeic acid, 2. Rosmarinic acid. 3. Astragaloside I, 4. Astragaloside II. The bands corresponding to the astragalosides are smaller than those in Figure 5. This is because a mixture of all astragalosides was analysed, resulting in coelution of I I II and II I IV.

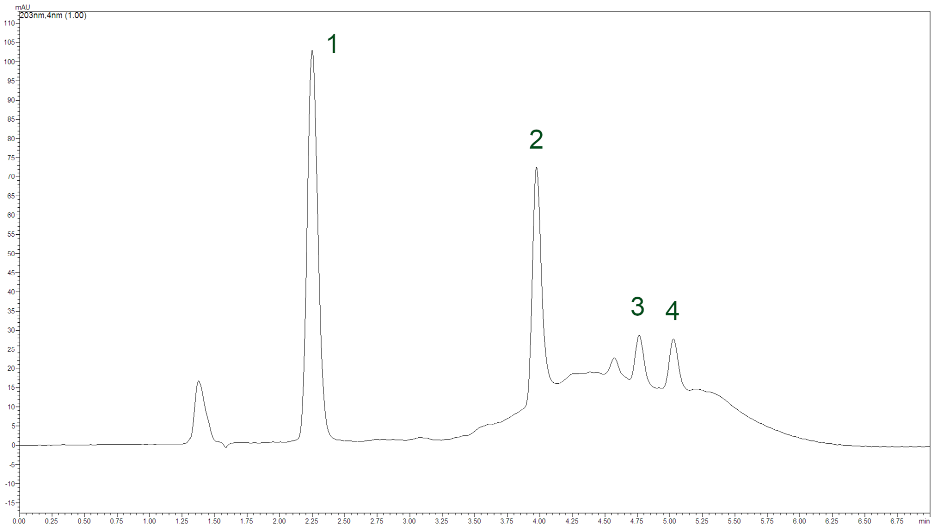

Figure S5. The chromatogram of a mixture of 1. Caffeic acid 2, Rosmarinic acid, 3. Astragaloside II, and 4. Astragaloside IV.

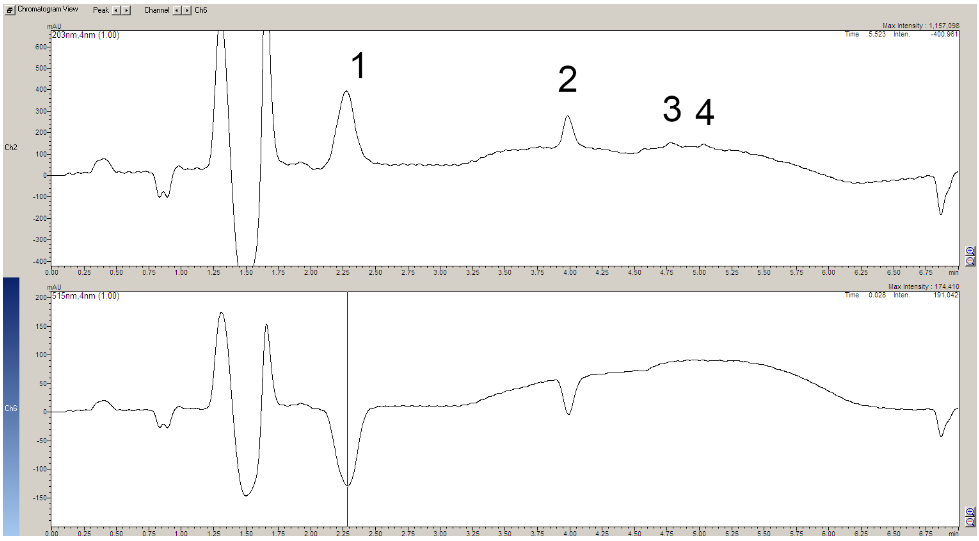

Figure S6. The chromatogram at 203 mn shows the separated compounds present in the methanolic extract of *A. mongholicus* roots along with additionally added caffeic acid and rosmarinic acid (top). The 515 nm chromatogram demonstrates the scavenging of the DPPH● radical by the separated compounds (bottom). Both chromatograms were saved in the same file. 1. Caffeic acid, 2. Rosmarinic acid. 3. Astragaloside II, 4. Astragaloside IV. The bands corresponding to the astragalosides are smaller than those in Figure 5. This is because a mixture of all astragalosides was analysed, resulting in coelution of I I II and II I IV.
